# Identification of *Ixodes ricinus* blood meals using an automated protocol with high resolution melting analysis (HRMA) reveals the importance of domestic dogs as larval tick hosts in Italian alpine forests

**DOI:** 10.1186/s13071-016-1901-y

**Published:** 2016-12-12

**Authors:** Margherita Collini, Francesca Albonico, Roberto Rosà, Valentina Tagliapietra, Daniele Arnoldi, Lorenza Conterno, Chiara Rossi, Michele Mortarino, Annapaola Rizzoli, Heidi Christine Hauffe

**Affiliations:** 1 Department of Biodiversity and Molecular Ecology, Research and Innovation Centre, Fondazione Edmund Mach, San Michele all’Adige, Trento, Italy; 2 Department of Veterinary Medicine, Università degli Studi di Milano, Milan, Italy; 3 Department of Food Quality and Nutrition, Research and Innovation Centre, Fondazione Edmund Mach, San Michele all’Adige, Trento, Italy

**Keywords:** *Ixodes ricinus*, Feeding ecology, Nymphs, Tick-borne disease, Blood meal analysis, Vertebrate hosts, *Canis lupus familiaris*

## Abstract

**Background:**

In Europe, *Ixodes ricinus* L. is the main vector of a variety of zoonotic pathogens, acquired through blood meals taken once per stage from a vertebrate host. Defining the main tick hosts in a given area is important for planning public health interventions; however, until recently, no robust molecular methods existed for blood meal identification from questing ticks. Here we improved the time- and cost-effectiveness of an HRMA protocol for blood meal analysis and used it to identify blood meal sources of sheep tick larvae from Italian alpine forests.

**Methods:**

Nine hundred questing nymphs were collected using blanket-dragging in 18 extensive forests and 12 forest patches close to rural villages in the Province of Trento. Total DNA was either extracted manually, with the QIAamp DNA Investigator kit, or automatically using the KingFisher™ Flex Magnetic Particle Processors (KingFisher Cell and Tissue DNA Kit). Host DNA was amplified with six independent host group real-time PCR reactions and identified by means of HRMA. Statistical analyses were performed in R to assess the variables important for achieving successful identification and to compare host use in the two types of forest.

**Results:**

Automating DNA extraction improved time- and cost-effectiveness of the HRMA protocol, but identification success fell to 22.4% (KingFisher™) from 55.1% (QIAamp), with larval hosts identified in 215 of 848 questing nymphs; 23 mixed blood meals were noted. However, the list of hosts targeted by our primer sets was extended, improving the potential of the method. Host identification to species or genus level was possible for 137 and 102 blood meals, respectively. The most common hosts were Rodentia (28.9%) and, unexpectedly, Carnivora (28.4%), with domestic dogs accounting for 21.3% of all larval blood meals. Overall, Cetartiodactyla species fed 17.2% of larvae. Passeriformes (14.6%) fed a significantly higher proportion of larvae in forest patches (22.3%) than in extensive forest (9.6%), while Soricomorpha (10.9%) were more important hosts in extensive forest (15.2%) than in forest patches (4.3%).

**Conclusions:**

The HRMA protocol for blood meal analysis is a valuable tool in the study of feeding ecology of sheep ticks, especially with the cost- and time- reductions introduced here. To our knowledge, we show for the first time that domestic dogs are important larval hosts in the Alps, which may have possible implications for tick-borne disease cycles in urbanized areas.

**Electronic supplementary material:**

The online version of this article (doi:10.1186/s13071-016-1901-y) contains supplementary material, which is available to authorized users.

## Background

The sheep tick *Ixodes ricinus* L. is the main European vector of a variety of pathogens of medical and veterinary importance [1–3]. This parasite takes blood meals on many wild and domestic vertebrate species (as well as humans), which may simply feed the tick (incompetent or maintenance hosts) or feed the tick and transmit the etiological agents of disease to the vector (competent hosts [4]). Thus, the epidemiology of tick-borne diseases (TBDs) depends on host, vector and pathogen dynamics, as well as on the complex network of interactions between them. Understanding tick feeding ecology in relation to the composition of vertebrate host communities is critical to predicting disease risk for public health and improving disease control strategies [5–8].

A blood meal is essential for the sheep tick to moult from one stage to the next, from larva to nymph to adult. Following the blood meal, the larvae and nymphs drop off the host and hide in the leaf litter for several months up to more than a year (depending on climatic conditions), before moulting to the next stage and waiting on the vegetation for the next host, which they detect using specialized sensors on their forelegs (‘questing’). Therefore, until recently*,* estimating host exploitation required capturing live hosts and counting feeding ticks, which is labour intensive and may be biased [9, 10].

Although molecular methods have been used successfully to identify host DNA in blood meals of several arthropod vectors, their application to questing ticks is more challenging since the blood meals will have been digested (and therefore degraded) for a year or more [6, 7, 11]. In fact, genetic blood meal identification for questing ticks with available methods was not considered sufficiently robust for application in field studies, and questions had been raised concerning their susceptibility to contamination [12, 13]. Recently, some of us developed an HRMA protocol to allow the identification of 21 of the most important vertebrate alpine hosts from field-collected questing sheep tick nymphs [11]. In this previous paper, we limited the sample size to the number of ticks necessary to provide proof of principle of the method. In the present study, we improved the time- and cost-effectiveness of this method with several modifications, and applied this new protocol to a large number of *I. ricinus* nymphs in order to evaluate tick host use in forest environments in the northeastern Italian Alps.

## Methods

### Tick sampling

**Fig. 1:**
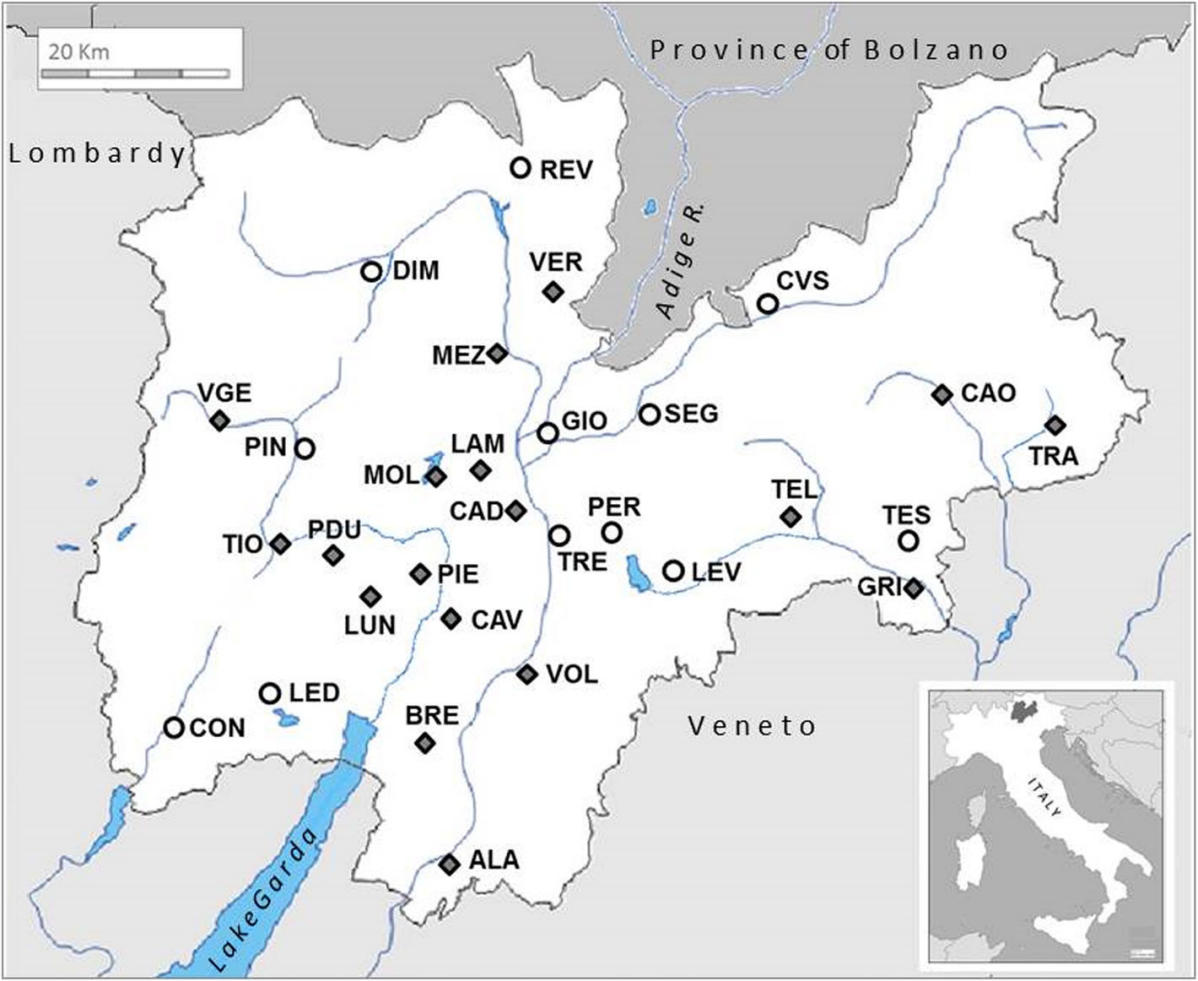
Location of sampling sites in the Province of Trento, northeastern Italy. Sampling site abbreviations are listed with the nearest locality to sampling site in Table 1. Open circles indicate sampling sites in forest patches (PATF) and *grey* diamonds indicate sites in or at the edge of extensive forests (EXTF)

From April to June 2012 and 2013, samples of 30 questing nymphs each were collected using standard blanket-dragging [14] from 30 deciduous and/or coniferous forest sites throughout the Province of Trento (Fig. 1; Table 1). Since forest extent has a significant effect on vertebrate community composition, the selected sites consisted of 18 extensive forests (EXTF) and 12 forest patches near human settlements (PATF). Humans typically visit extensive forest (EXTF) for work-related activities (e.g. rangers, lumberjacks, farmers, hunters) and forest patches (PATF) closer to villages for leisure activities (dog walking, hiking, fishing, mushrooming). Each tick was removed individually from the blanket using sterile forceps, placed in a sterile 2 ml microcentrifuge tube, transported live to the Fondazione E. Mach laboratories and frozen at -80 °C until DNA extraction.

**Table 1:** Sampling sites, collection data and HRMA blood meal analysis results according to DNA extraction method

DNA extraction method	Sampling site^a^	Nearest locality to sampling site	Sampling time	% identification success^b^	No. of mixed blood meals	Mean identification success (%)
QiaAMP® DNA Investigator	BRE^c^	Brentonico	May 2012	46.7 (7/15)	1	55.1
	CAD^c^	Cadine	May 2012	35.7 (5/14)	0	
	CON	Condino	April 2012	69.2 (9/13)	4	
	GIO^c^	Giovo	April 2013	43.8 (7/16)	1	
	GRI^b^	Grigno Valsugana	April 2013	31.3 (5/16)	0	
	PIE	Pietramurata	May 2012	92.3 (12/13)	2	
	TRA	Transacqua	June 2012	66.7 (8/12)	2	
ThermoScientific KingFisher^TM^	ALA	Ala	May 2012	33.3 (10/30)	2	22.4
	BRE^c^	Brentonico	May 2012	29.4 (5/17)	0	
	CAO	Caoria	April 2013	30.0 (9/30)	2	
	CAD^c^	Cadine	May 2012	25.0 (2/8)	0	
	CAV	Cavedine	April 2013	20.0 (6/30)	0	
	CVS	Cavalese	May 2012	21.7 (5/23)	0	
	DIM	Dimaro	May 2013	33.3 (10/30)	2	
	GIO^c^	Giovo	April 2013	31.3 (5/16)	0	
	GRI^c^	Grigno Valsugana	April 2013	43.8 (7/16)	0	
	LAM	Laghi di Lamar	May 2012	9.7 (3/31)	0	
	LED	Ledro	May 2012	20.0 (6/30)	0	
	LEV	Levico	April 2012	26.7 (8/30)	0	
	LUN	Lundo	May 2012	6.7 (2/30)	0	
	MEZ	Mezzocorona	June 2012	16.7 (5/30)	2	
	MOL	Molveno	May 2012	3.3 (1/30)	0	
	PDU	Passo del Durone	June 2012	32.4 (11/34)	0	
	PER	Pergine	April 2012	20.0 (6/30)	1	
	PIN	Pinzolo	May 2012	20.0 (6/30)	0	
	REV	Revò	May 2012	13.3 (4/30)	0	
	SEG	Segonzano	May 2012	23.3 (7/30)	1	
	TEL	Telve	June 2012	13.3 (4/30)	1	
	TES	Tesino	June 2012	20.0 (6/30)	0	
	TIO	Tione di Trento	May 2012	10.0 (3/30)	0	
	TRE	Trento	May 2013	17.6 (6/34)	0	
	VER	Vervò	May 2012	20.0 (6/30)	0	
	VGE	Val Genova	May 2013	13.3 (4/30)	0	
	VOL	Volano	May 2012	50.0 (15/30)	2	

^*a*^ *Abbreviations* as in Fig. 1

^b^Number of nymphs with identified blood meal/number of nymphs analysed (as shown in parentheses)

^c^Site with samples extracted with either QiaAMP® DNA Investigator or ThermoScientific KingFisher^TM^ methods

### DNA extraction and larval blood meal source identification by real-time HRMA

Each tick was confirmed morphologically as *I. ricinus* using a dissecting microscope at 50× magnification following Cringoli et al. [15] then washed twice in DNA-free distilled water to eliminate surface contaminants. For 99 nymphs (including 52 used in [11]), DNA was extracted manually using the QiaAmp® DNA Investigator Kit (Purification of total DNA from nail clipping and hair Protocol; Qiagen, Valencia, CA, USA) following Collini et al. [11]. For the remaining 749 nymphs, blood meal identification also followed Collini et al. [11] except that total DNA extraction was performed automatically using the KingFisher™ Flex Magnetic Particle Processors with the KingFisher Cell and Tissue DNA Kit (Thermo Fisher Scientific, Vantaa, Finland), in an attempt to improve speed and efficiency. Sample lysis was performed under a biological laminar flow hood (UV-sterilized) as follows: a single nymph was placed in a sterile 2 ml microcentrifuge tube containing 180 μl ATL buffer (Qiagen, Valencia, CA, USA), 30 μl DTT 1 M (Sigma-Aldrich, Saint Louis, USA) and 20 μl Proteinase K (KingFisher™ Cell and Tissue DNA Kit; Thermo Fisher Scientific, Vantaa, Finland), and cut into small pieces with a sterile scalpel. Samples were digested overnight by incubating at 56 °C. Purification followed the manufacturer’s protocol, except that total DNA was eluted in 80 μl of elution buffer (Thermo Fisher Scientific) instead than 150 μl. DNA was stored at -20 °C until use. To check for cross-contamination, three negative controls were included in each 96-well extraction plate. DNA of target host species (positive controls for real-time HRMA) was extracted from host tissues and/or from engorged ticks collected from the host while feeding using the methods above (these samples were available from previous or ongoing projects at the Fondazione Edmund Mach, Italy, and stored in 70% ethanol solution at -80 °C). No tissues or engorged ticks were available for *Sorex araneus* or *Crocidura russula*.

Host DNA from the larval blood meal remnant in questing nymphs was amplified using group-specific primers and reaction mix conditions as already described [11]. In each real-time HRMA reaction, a positive control sample for each target species and a negative control (DNA-free water) were included. A QIAgility robotic workstation (Qiagen, Valencia, CA, USA) was used for automated high precision reaction setup. Real-time HRMA was performed in a Rotor-Gene^TM^ 6000 real-time cycler (Corbett Life Science) with a 72-well rotor, according to Collini et al. [11] with the following modifications: initiation step 95 °C for 5 min, 55 annealing and elongation cycles at 95 °C for 15 s and T_*a*_ (°C) of the group-specific primer set for 15 s, directly followed by HRM with a pre-melt conditioning step of 70 °C for 90 s, then increasing the temperature from 70 °C to 95 °C, by 0.2 °C steps for 2 s each. The Rotor-Gene 6000 Series Software v. 1.7 was used to control amplifications and to perform HRMA to identify the blood meal source by means of both normalized and derivative melting profile shapes and melting temperatures (*T*_*m*_ °C), as described in Collini et al. [11]. To check that the DNA extraction protocol modifications and change of real-time HRM instrument did not introduce errors in blood meal identification, the first few amplified fragments from questing nymphs were sequenced; subsequently, since there were no errors in blood meal identification, only amplicons with non-specific melting properties were investigated *via* capillary electrophoresis and/or sequence analysis. Capillary electrophoresis was carried out using the QIAxcel system (Qiagen, Valencia, CA, USA) with a DNA High Resolution Cartridge and the QX 15 bp-3 Kb size marker, using the OM500 method; results were analysed with QIAxcel ScreenGel 1.0.2.0. For sequencing, real-time PCR products were purified with Exo-SAP-IT™ (GE Healthcare, Little Chalfont, England); both forward and reverse strands were sequenced on an ABI 3130 XL using Big Dye Terminator v3.1 (Applied Biosystems, Foster City, CA, USA). Raw sequences were checked and a consensus sequence created using the software Sequencher v. 5.1; a BLASTn search (http://blast.ncbi.nlm.nih.gov/Blast.cgi) was carried out to verify amplicon identity.

### Statistical analysis

A Multiple Linear Regression Model was used to assess variation in the proportion of ticks with an identified blood meal (identification success) in relation to the following explanatory variables: DNA extraction method, sampling year, sampling month and habitat type. Tick hosts were grouped according to taxonomic order (Rodentia, Soricomorpha, Passeriformes, Carnivora, Cetartiodactyla) and a chi-square test was used to compare the proportion of identified blood meals in the different host groups between EXTF and PATF sites. Statistical analyses were performed using R software [16].

## Results

Out of the 900 sheep tick nymphs collected from 30 sites in Province of Trento, DNA was extracted from 848 (506/540 ticks collected in EXTF and 342/360 in PATF). Larval blood meals were identified in 215 of these (25.4% identification success; Table 3). Identification success varied widely between sampling sites (from 3.3 to 92.3%; Table 1). The automated KingFisher™ technology tested here on 749 nymphs allowed DNA extraction from 96 samples per day by two operators, compared to 12 per day with QiaAmp® DNA Investigator method, as well a two-thirds decrease in reagent cost. In addition, the robotic preparation of the PCR reactions decreased working time and possible errors associated with manual set-up.

**Table 2:** Linear model results for blood meal identification success. Reference levels for the explanatory variables are QIAamp for ‘Method’, 2012 for ‘Year’, April for ‘Month’ and EXTF for ‘habitat type’

Explanatory variable	Coefficient	SE^a^	*t*-value^b^	Pr(>|t|)^c^
(Intercept)	58.62	7.82	7.44	< 0.001
Method_ KingFisher™	-31.76	6.37	-4.99	< 0.001
Year_2013	-3.21	6.33	-0.51	0.62
Month_May	-4.28	7.01	-0.61	0.55
Month_June	-4.74	9.29	-0.51	0.61
Habitat type_PATF	-0.38	5.29	-0.07	0.94

^a^Standard error of estimated coefficient

^b^ *t*-value estimate to standard error ratio

^c^Probability for *t*-value

The linear model identified DNA extraction method as the only factor that significantly affected identification success (t-value = -4.99, *df* = 28, *P* = 2.87e^-5^; Table 2). Hosts could be identified from tick nymphs 55.1% of the time if total DNA was extracted with QiaAmp® DNA Investigator method compared to 22.4% identification success for total DNA extracted using the KingFisher™ Cell and Tissue DNA kit (Table 1). It should be noted that the amplicons from host DNA extracted from questing nymphs with the KingFisher™ kit demonstrated a higher *T*_*m*_ than those extracted with the QiaAmp® DNA Investigator kit. If melting pattern was not sufficient to confirm a shift in *T*_*m*_ *,* sequencing was used to confirm the host species corresponding to the amplicons; the new melting temperatures were then used as reference *T*_*m*_ (Additional file 1: Table S1).

The identification of the host species providing a larval blood meal was possible in 137 cases, while 102 hosts were identified to genus (67 for *Apodemus* spp.; nine for *Sorex* spp.; 19 for *Turdus* spp./*E. rubecula* and seven for *Ovis* spp.), as reported in Table 3. As explained in Collini et al. [11], HRMA could not reliably discriminate between *T. philomelos* and *E. rubecula* control samples as a result of overlapping *T*_*m*_. In addition, when amplicons with a lower *T*_*m*_ (84.10 °C, 84.14 °C and 84.24 °C) than usual for *T. philomelos/E. rubecula* (84.44–84.50 °C) were sequenced, BLASTn could only identify them as *Turdus* spp. or *T. philomelos*; therefore, amplicons having a *T*_*m*_ in the range 84.00 to 84.50 °C were all classified as *Turdus* spp./*E. rubecula* (Table 1). We also showed here that the Cervidae primer set amplifies a wider range of Cetartiodactyla hosts than predicted, including some species already targeted by the Caprinae primers (*Ovis* spp. and *R. rupicapra*), as well as fallow deer (*Dama dama*)*;* however, sequence analysis of *Ovis* amplicons obtained with Cervidae primers indicated that HRMA of the control region fragment could not discriminate domestic (*O. aries*) and wild (*O. aries musimon*) sheep (BLASTn reports 99–100% identity scores for *O. aries, O. a. musimon* and *O. orientalis*; see Additional file 2: Table S2). Consequently, all sequences BLASTn matched with various sheep species or subspecies with a similar identity scores were classified as *Ovis* spp. Because *T*_*m*_ of several species of Cervidae overlapped*,* identification by HRMA also became complex and all amplicons were sequenced to confirm species (Additional file 1: Table S1). In addition, HRMA with the Muroidea primer set led to the misidentification of five *Apodemus* spp. blood meals as *M. glareolus*, because of intraspecific variation caused by 2 to 9 nucleotide mutations (Additional file 3: Figure S1). A single blood meal from a Chinese hamster (*Cricetus griseus*) was also amplified using the Muroidea primers, producing an amplicon with a *T*_*m*_ overlapping with that of *Apodemus* spp (82.40 °C). We assume this blood meal originated from a companion animal since *C. griseus* does not occur naturally in Europe (such a result is unlikely to be due to contamination since samples of this species had not been processed previously in our laboratory).

**Table 3:** Blood meal identification in questing nymphs from EXTF and PATF in the Province of Trento, Italy

Larval hosts targeted (primer set used)^a^	Number and percentage of identified hosts in questing nymphs		
	Forest type		Total (%)^h^
	EXTF (%)^h^	PATF (%)^h^	
Rodentia (Muroidea)	46 (31.7)	23 (24.5)	69 (28.9)
*Apodemus* spp.	44 (30.3)	23 (24.5)	67 (28.0)
*Myodes glareolus*	1 (0.69)	0	1 (0.4)
*Mus musculus*	0	0	0
*Cricetus griseus* ^b^	1 (0.69)	0	1 (0.4)
Carnivora (Canidae)	33 (22.7)	35 (37.2)	68 (28.4)
*Canis lupus familiaris*	28 (19.3)	23 (24.5)	51 (21.3)
*Vulpes vulpes*	5 (3.4)	12 (12.7)	17 (7.1)
Cetartiodactyla (Caprinae, Cervidae)	30 (20.7)	11 (11.7)	41 (17.2)
*Ovis* spp.^c,d^	5 (3.4)	2 (2.1)	7 (2.9)
*Rupicapra rupicapra* ^d^	3 (2.1)	0	3 (1.3)
*Capra hircus* ^c^	2 (1.4)	0	2 (0.8)
*Bos taurus* ^c^	0	3 (3.2)	3 (1.3)
*Capreolus capreolus* ^d^	8 (5.5)	2 (2.1)	10 (4.2)
*Cervus elaphus* ^d^	12 (8.3)	3 (3.2)	15 (6.3)
*Dama dama* ^*b*,d^	0	1 (1.1)	1 (0.4)
Passeriformes (Passeriformes)	14 (9.6)	21 (22.3)	35 (14.6)
*Turdus merula*	8 (5.5)	8 (8.5)	16 (6.7)
*Turdus* spp*./Erithacus rubecula* ^e^	6 (4.1)	13 (13.8)	19 (7.9)
Soricomorpha (Soricidae)	22 (15.2)	4 (4.3)	26 (10.9)
*Sorex* spp.	9 (6.2)	0	9 (3.8)
*Sorex araneus*	0	0	0
*Crocidura leucodon*	7 (4.8)	2 (2.1)	9 (3.8)
*Crocidura suaveolens*	6 (4.1)	2 (2.1)	8 (3.3)
*Crocidura russula*	0	0	0
Total no. of nymphs analysed	506	342	848
Total no. of nymphs with identified blood meal	130	85	215
Total no. of hosts identified	145	94	239
% identification success^f^	25.7	24.9	25.4
No. of mixed blood meals identified	14	9	23
% of mixed blood meals^g^	10.8	10.6	10.7

*Abbreviations*: *EXTF* extensive forest, *PATF* patchy forest

^a^Host group primers as described in Collini et al. [11]

^b^Host not targeted in original protocol as described by Collini et al. [11], but identified here after HRMA, by sequencing and BLASTing

^c^Amplified with Caprinae primers

^d^Amplified with Cervidae primers

^e^HRMA of Passeriformes amplicons did not allow discrimination between *T. philomelos* and *E. rubecula*

^f^Total no. of nymphs with identified blood meal/total no. of nymphs analysed * 100

^g^Total no. of mixed blood meals identified/total no. of nymphs with identified blood meal * 100

^h^No. of identified host for taxonomic level/total no. hosts identified * 100

Our results show that in Trentino, the most common larval hosts were Rodentia (28.9%), mainly *Apodemus* spp. (28.0%). The second most frequent host group was Carnivora (28.4%), with *C. l. familiaris* accounting for 21.3% and *V. vulpes* for 7.1%. Cetartiodactyla species fed 17.2% of larvae, with *C. elaphus* and *C. capreolus* being the most common hosts (6.3% and 4.2%, respectively). The 14.6% of identified blood meals belonged to Passeriformes and, lastly, 10.9% of blood meals were derived from Soricomorpha. Of the entire list of target hosts, only *C. russula*, *S. araneus* and *Mus musculus domesticus* did not appear to be larval blood meal sources in the study area (Table 3).

**Fig. 2:**
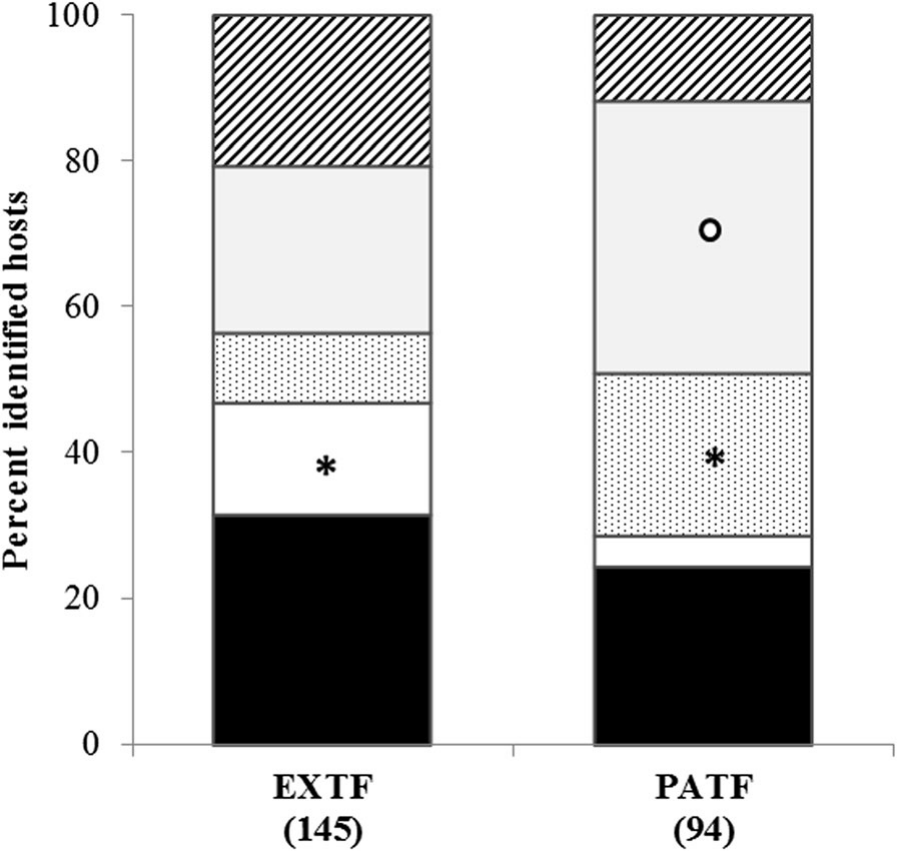
Percent of *I. ricinus* larvae hosts from the five taxonomic orders, according to habitat type: Cetartiodactyla (*diagonal stripes*), Carnivora (*light grey*), Passeriformes (*stippled*), Soricomorpha (*white*), Rodentia (*black*). Chi-square test: *P < 0.05; oP < 0.10

Blood meal identification success was similar in EXTF (130/506; 25.7%) and PATF (85/342; 24.9%) sites (Table 3). The proportion of Soricomorpha blood meals was significantly higher in EXTF than in PATF (15.2 *vs* 4.3%, respectively; *χ* ^2^ = 5.03, *df* = 1, *P* = 0.014), while the opposite was true for Passeriformes (PATF: 22.3% and EXTF: 9.6%; *χ* ^2^ = 4.29, *df* = 1, *P* = 0.024) and, at the limit of significance, for Carnivora (PATF: 37.2% and EXTF: 22.8%, respectively; *χ* ^2^ = 2.99, *df* = 1, *P* = 0.063), as presented in Fig. 2. No significant differences were observed between the proportion of Rodentia (EXTF = 31.7%; PATF = 24.5%; *χ* ^2^ = 0.68, *df* = 1, *P* = 0.337) or Cetartiodactyla (EXTF = 20.7%; PATF = 11.7%; *χ* ^2^ = 1.97, *df* = 1, *P* = 0.114) acting as larval hosts in the two forest habitats (Fig. 2).

Interestingly, DNA from multiple hosts was recovered simultaneously from 23 nymphs (10.7%; Tables 1, 4). All mixed blood meals consisted of DNA from two hosts, except one for which three hosts (*Apodemus* spp., *Turdus* spp.*/E. rubecula* and *Ovis* spp.) were identified. The most prevalent host in this group of blood meals, *Apodemus* spp., was found in association with eight other hosts in 13 nymphs; the second most prevalent, *C. l. familiaris*, was found together with 4 different hosts in 9 nymphs (Table 4). It should be noted that DNA from multiple hosts in single nymphs was obtained by independent amplifications with the different primer sets, supporting the reliability of their identification.

**Table 4:** Number of mixed larval blood meals with specific host compositions

	*Apodemus* spp.	*Sorex* spp.	*Vulpes vulpes*	*Capreolus capreolus*	*Cervus elaphus*	*Bos taurus*
*Apodemus* spp.			1		2	
*Myodes glareolus*				1		
*Crocidura leucodon*			2		1	
*Crocidura suaveolens*	1					
*Turdus merula*	1					
*Turdus* spp.*/Erithacus rubecula*	2^a^		1			
*Canis lupus familiaris*	4	1			1	3
*Ovis* spp.	1^a^					
*Capra hircus*	1					

^a^From a single nymph, DNA from *Apodemus* spp., *Turdus.* spp.*/E. rubecula* and *Ovis* spp. was identified

## Discussion

This study shows that the real-time HRMA protocol for blood meal analysis in questing tick nymphs of Collini et al. [11] is a reliable method for the identification of at least 20 larval host species and genera. Moreover, the introduction of the automated DNA extraction method and the robotic preparation of PCR reactions improved the time- and cost-effectiveness of the HRMA protocol for blood meal analysis presented here. However, there was a notable decrease in the identification success noted here (25.4% overall) compared to the 65.4% reported in Collini et al. [11] based on manual DNA extraction and PCR set-up only, and to other blood meal analysis methods (RLBH: 26.4% [17], 53.1% [18], 49.4% [19], 33.0% [20], 43,6% [21], 62.8% [22]; RFLP: 62.8% [23]). We assume this decrease was a direct result of the DNA extraction method, although site-specific climatic conditions may also have had an effect, as noted by Morán Cadenas et al. [21]. Unfortunately, we do not have the relevant measurements at our sample sites of the most important climatic variables regulating tick activity, such as saturation deficit [24], which would have allowed an investigation of this effect.

Here we also extended the list of hosts detected by the primer sets for the Cervidae (from two to five with the addition of *D. dama*, *Ovis* spp. and *R. rupicapra*) and Passeriformes (from two to three with the addition of *Turdus* spp.). On one hand, extending the host species list improves the accuracy of the method in reliably assessing host richness; on the other, since primer targets within host groups have relatively low levels of variability, the *T*_*m*_ overlapped for some species, and therefore amplicons needed to be sequenced to confirm species identification. Sequencing could be avoided if identification of host group or genus is adequate for answering the scientific question of the study; for example, our results showed that grouping hosts by Order still gave important insight into larval host distribution in the habitats investigated here. However, possible solutions exist to enhance species-level identification, such as the use of unlabeled probes [25] or a secondary HRMA on the heteroduplexes formed between the reference and the unknown amplicons [26]. It might be particularly interesting to discriminate between blood meals derived from wild and domestic sheep, since it was recently noted that wild relatives of domesticated species may be unrecognized reservoirs of zoonotic pathogens (see [3] and references therein; [27]). Since mutations can also confound species identification in the Muroidea, the above suggestions may prove useful for identifying host species in this group as well.

Despite the problems encountered above, we believe that this method, which eliminates contamination, a troublesome feature of previous methods, is still a valuable tool in the study of the feeding ecology of hard ticks. A variety of possibilities still exist for its improvement, from the DNA extraction method to host identification. For example, considering the importance of lizards in some TBDs, the panel of targeted hosts could be enhanced accordingly as a future improvement of the HRMA assay.

The application of the HRMA protocol to a large number of tick nymphs from across the Province of Trento has lent insight into the feeding ecology of larval ticks, and TBDs risk in this area. As forests cover over 50% of the province, villages and other residential areas are embedded in a rural ecosystem where work and leisure activities promote close contact with tick habitat. In addition, local wildlife includes many of the species recognized as competent reservoirs for zoonotic pathogens*,* as well as tick spreaders and maintenance hosts [3, 28]. Our data confirmed that *Apodemus* spp. are the most important larval hosts, providing 28.0% of blood meals, while passerine birds accounted for another 14.6%. With rodent meals and, less frequently, bird blood meals, larval ticks may acquire pathogens such as *B. burgdorferi* (*s.l*.), TBEv and *Anaplasma phagocytophilum* [3, 29–33]. The pathogens multiply in the salivary glands of the tick and may be transmitted to the next host, including humans, establishing the basis for disease hazard as a result of infected tick presence in both forest habitats [8]. In addition, ungulates like *C. capreolus* and *C. elaphus* are known to be critical to the maintenance of the sylvatic cycle of these pathogens, as they feed ticks at various stages [33, 34]. Our analyses indicate that species of Cetartiodactyla are important larval hosts in both PATF and EXTF (17.2% overall), with *C. capreolus* and *C. elaphus* being the most represented (4.2 and 6.3% respectively), meaning that TBDs cycles are well-supported across the study area.

This is the first blood meal study to consider domestic dogs as larval tick hosts. Unexpectedly, our results show that species of Carnivora are as important as rodents in providing larval blood meals (28.4%) in the study area. Of the 51 amplicons identified by HRMA as *C. l. familiaris*, 19 were sequenced and all of them identified as *C. l. familiaris* with a 99–100% of identity score by BLASTn (https://blast.ncbi.nlm.nih.gov/Blast.cgi). Additionally, as reported in Additional file 1: Table S1, HRMA discrimination between *C. l. familiaris* and *V. vulpes* was straightforward thanks to their particular discriminant melting temperatures and to differences in the number of peaks (a single peak for dogs and two peaks for foxes). As for other wild European carnivores (badger *Meles meles* and raccoon dog *Nyctereutes procyonoides* [35]; marbled polecat *Vormela peregusna* and European mink *Mustela lutreola* [36]), the role of dogs and foxes in the epidemiology of TBDs is still not well-defined as they do not appear to be amplification hosts for TBEv [37], or reservoir hosts for *Borrelia burgdorferi* (*s.l*.) [38] or *A. phagocytophilum* [28, 39], although all of these mammal species are known to carry these pathogens. Most importantly for human tick bite and TBD risk, dogs appear to be important hosts in both EXTF and PATF. This means they contribute to maintaining TBD cycles as tick feeders in both habitats, but could also carry infected ticks from EXTF and/or PATF into urban parks, gardens and houses where they may later drop off and parasitize a variety of urban-adapted vertebrate tick hosts including humans [3, 38, 40–43]. The potential impact of domestic dogs and foxes on TBD cycles definitely deserves further attention. In addition, dogs may represent a target for tick population control in urbanized areas, and could act as sentinels for TBDs [38, 44].

It has recently been suggested that domesticated hosts, such as dogs (but also cats, cattle, sheep and goats), are potential tick and pathogens super-spreaders, connecting epidemiological systems involving different tick species, vertebrate hosts and pathogens [4]. Our study confirms that larval *I. ricinus* feeds equally on many wild and domesticated hosts in the same habitat; moreover, in the same study area, tick species other than *I. ricinus* have been found feeding on dogs and birds [45]. Since our study also shows that ground-feeding passerine species are more important larval hosts in PATF than EXTF, being common in urban green areas, we would add that these Passeriformes, together with foxes, could also be considered additional spreader species. Only Soricomorpha, that are generally negatively affected by habitat fragmentation and agricultural activities [46, 47], are more represented in EXTF, and are unlikely to act as bridge species; however, as they feed more than 10% of tick larvae and given their recognized reservoir status for some pathogens, their role in the wild TBD cycles should not be underestimated [48].

DNA of more than one host was detected in 10.7% of single nymphs, in agreement with previous studies [21, 22, 49]. While it has been suggested that mixed blood meals are the result of contamination, this is unlikely in our study since all DNA extraction and amplification controls were negative. In addition, mixed blood meals were unambiguous, as hosts were identified in independent reactions with different host-group specific primer sets. Although multiple host blood meals run contrary to the widely held view that *I. ricinus* takes one blood meal from a single host per life stage (see Background), Gray et al. [49] also observed the occasional collection of semi-engorged larvae by blanket dragging, suggesting that multiple blood meals are occasionally necessary for full engorgement, after voluntary drop off, involuntary interrupted feeding, or unsuccessful full attachment. We observed that many different host combinations are possible (see Table 4): two rodents, rodent and bird, rodent and carnivore, rodent and ungulate, etc., presumably not only resulting in mixed blood meals, but also promoting multiple pathogen transmission between hosts. This phenomenon deserves a more in depth analysis, especially in view of the fact that pathogen co-infections in questing ticks at both nymph and adult stages have also been widely observed [18–20, 50–53], and notably, such multiple infections from a single tick bite in humans have clinical symptoms showing complex patterns making diagnosis and prophylaxis challenging [54, 55]. Multiple infections have been explained up to now by transtadial/transovarial pathogen transmission and acquisition of additional pathogens by successive meals taken at different stages [52, 53]; however, a significant source of such co-infection could derive from repetitive blood meals at the same stage.

## Conclusions

The HRMA protocol of Collini et al. [11] is a reliable method for larval blood meal analysis in field-collected sheep tick nymphs, although identification success is lower if DNA extraction is automated and sequencing of amplicons is still necessary for species confirmation with several primer sets. The results obtained from 848 ticks from 30 sites across the Province of Trento show that rodents and wild ungulates are widely exploited as larval blood meal sources, supporting TBDs sylvatic cycles across the study area. In addition, we have also shown for the first time that domestic dogs are important larval tick hosts, with possible implications not only for tick population maintenance and spread of TBD in urban and semi-urban areas, but also for tick control strategies.

## Additional files

Additional file 1: Table S1. Melting temperatures for amplicons obtained from questing tick extracted using the KingFisher Cell and Tissue kit. (PDF 415 kb)
Additional file 2: Table S2. BLASTn search results and alignment for *Ovis* spp. amplicons obtained with Cervidae primer set. (PDF 323 kb)
Additional file 3: Figure S1. Sequence alignment of mtDNA *control region* amplicons generated with the Muroidea primer set. (PDF 307 kb)

## Abbreviations

DNA: Deoxyribonucleic acid
EXTF: Extensive forest
HRMA: High resolution melting analysis
mtDNA: Mitochondrial DNA
PATF: Forest patches surrounding human settlements
PCR: Polymerase chain reaction
RFLP: Restriction length fragment polymorhisms
RLBH: Reverse line blot hybridization
*T*_*m*_: Melting temperature
TBD: Tick-borne disease
