# Supplementary material for: Identification of Ixodes ricinus blood meals using an automated protocol with high resolution melting analysis (HRMA) reveals the importance of domestic dogs as larval tick hosts in Italian alpine forests
Source: Parasit Vectors. 2016 Dec 12;9:638. doi: 10.1186/s13071-016-1901-y (PMC5154095; doi:10.1186/s13071-016-1901-y)
Supplement: Additional file 1: Table S1. — Melting temperatures for amplicons obtained from questing tick extracted using the KingFisher Cell and Tissue kit. (PDF 415 kb) [file 13071_2016_1901_MOESM1_ESM.pdf]

Additional file 1.

Table: melting temperatures for amplicons obtained from questing ticks DNA extracted using the KingFisher Cell and Tissue kit. $\boldsymbol{T_m}$**1**. melting temperature peak 1; $\boldsymbol{T_m}$**2**. melting temperature peak 2 (if present). In bold are newly added hosts species for the primer set, identified by sequencing because of deviating melting pattern.

**Host-group primer set**	**Amplified host species**	$\boldsymbol{T_m}$**1**			$\boldsymbol{T_m}$**2**		
		**min**	**max**	**mean**	**min**	**max**	**mean**
**Canidae**	*C. l. familiars*	80.86	81.56	81.4	-	-	-
	V. vulpes	81.34	82.04	81.68	83.54	84.2	83.74
**Cervidae**	*C. capreolus*	81.36	81.76	81.59	83	83.5	83.21
	C. elaphus	81.5	82.44	81.86	83.74	84.2	84
	***Ovis* sp.**	81.96	82.16	82.05	83.4	83.9	83.75
	D. dama	na	na	81.66	na	na	83.64
	R. rupicapra	81.8	81.9	81.85	83.5	83.64	83.57
**Passeriformes**	*T. merula*	84.2	85.34	84.9	-	-	-
	T. philomelus/ E. rubecula	84.00	84.50	84.24	-	-	-
	***Turdus* spp.**	84.10	84.24	84.17	-	-	-
**Soricidae**	*Sorex* sp.	81.16 (80.5*)	81.44	81.32°	-	-	-
	C. leucodon	na	na	82.70	-	-	-
	C. suaveolens	81.10	81.20	81.15	-	-	-
**Muridea**	*Apodemus* sp.[#]	82.2	82.84	82.37	-	-	-
	C. griseus	na	na	82.40			
**Caprinae**	*O. aries*	na	na	77	na	na	82.9
	R. rupicapra	na	na	77.1	na	na	81.1
	C. hircus	77.9	78	77.95	-80.86	-80.9	80.99

* a single amplicon showed this low T_m.
° the lowest Tm was not used to compute the mean T_m.
[#] deviating melting temperature for *Apodemus* amplicons with deletions and mutated positions are not included in that calculation of T_m (see Additional file 3).
