# Supplementary material for: Identification of Ixodes ricinus blood meals using an automated protocol with high resolution melting analysis (HRMA) reveals the importance of domestic dogs as larval tick hosts in Italian alpine forests
Source: Parasit Vectors. 2016 Dec 12;9:638. doi: 10.1186/s13071-016-1901-y (PMC5154095; doi:10.1186/s13071-016-1901-y)
Supplement: Additional file 2: Table S2. — BLASTn search results and alignment for Ovis spp. amplicons obtained with Cervidae primer set. (PDF 323 kb) [file 13071_2016_1901_MOESM2_ESM.pdf]

Additional file 2.

Table: BLASTn search results for HRM_Cer *Ovis* spp. amplicons, showing the lack of a reliable amplicon species/subspecies identity assignment given the high (100-99%) and equal identity score resulting for different *Ovis* spp..

**HRM_Cer amplicon from engorged female from mouflon**	BLASTn: Sequences producing significant alignments	Max score	Total score	Query cover	e-value	Ident %
IRH000213	*Ovis orientalis* breed Asian mouflon	302	302	100	1E-78	100
	Ovis aries breed Jingzhong	302	302	100	1E-78	100
	Ovis aries	302	302	100	1E-78	100
	Ovis aries	302	302	100	1E-78	100
	Ovis aries musimon	302	302	100	1E-78	100
**HRM_Cer amplicons from questing nymphs**						
IRQ52013_Mez	*Ovis aries musimon*	300	300	100	5E-78	100
	Ovis aries	298	298	99	2E-77	100
	Ovis aries	298	298	99	2E-77	100
	Ovis aries	298	298	99	2E-77	100
	Ovis orientalis breed Asian mouflon	294	294	100	2E-76	99
IRQ52513_Mez	*Ovis orientalis* breed Asian mouflon	303	303	100	4E-79	100
	Ovis aries breed Jingzhong	303	303	100	4E-79	100
	Ovis aries	303	303	100	4E-79	100
	Ovis aries	303	303	100	4E-79	100
	Ovis aries musimon	303	303	100	4E-79	100
IRQ079212_A_Ala	*Ovis orientalis* breed Asian mouflon	296	296	100	6E-77	99
	Ovis aries breed Jingzhong	296	296	100	6E-77	99
	Ovis aries	296	296	100	6E-77	99
	Ovis aries	296	296	100	6E-77	99
	Ovis aries musimon	296	296	100	6E-77	99
IRQ15013_Vge	*Ovis orientalis* breed Asian	302	302	100	1E-78	100
	Ovis aries breed Jingzhong	302	302	100	1E-78	100
	Ovis aries	302	302	100	1E-78	100
	Ovis aries	302	302	100	1E-78	100
	Ovis aries musimon	302	302	100	1E-78	100
IRQ059412_Pin	*Ovis ammon hodgsoni*	303	303	100	4E-79	100
	Ovis aries ophion	298	298	100	2E-77	99
	Ovis orientalis breed Asian mouflon	298	298	100	2E-77	99
	Ovis aries breed Jingzhong	298	298	100	2E-77	99
	Ovis aries	298	298	100	2E-77	99
	Ovis orientalis anatolica	298	298	100	2E-77	99

According toTaxonomy and Genbank NCBI database, the following synonyms refer to mouflon (*Ovis aries musimon* Pallas, 1811): *Ovis orientalis musimon*; *Ovis aries mufflon*; *Ovis musimon*; *Ovis gmelini*; *Ovis ammon musimon*.

Alignment (next page): *Ovis* spp. mtDNA *control region* sequences retrieved from GenBank (BG-1 to 13), with the corresponding GenBank taxonomic identity, and *Ovis* spp. amplicons generated with the primer set HRM_ Cer (IRH000213 amplicon from engorged tick collected from a wild sheep; IRQxxxxx amplicons from questing nymphs), together with one *Capreolus capreolus* (IRQ02713) and one *Cervus elaphus* (IRQ039412) amplicons from questing nymphs obtained in this study.
The alignment shows the lack of mutation in the amplified fragment such as to permit the discrimination between *Ovis aries* and its subspecies, justifying the BLASTn search results reported in the previuos Table. Additionally, the alignment underline the high intraspecific variability in *Ovis aries* and the lack of defined interspecific mutations compared to *C. capreolus* and *C. elaphus*, explaning the complexity in HRMA amplicons identification with the HRM_Cer primer set.

```
GB-1_O.aries_Z35264             CGATGGACTAATGACTAATCAGCCCATGCCTA-ACATAACAGTGGTGTCATGCATTTGGTATTTTTTAAT
GB-2_O.aries_Z35258             ................................-.....................................
GB-3_O.aries_musimon_GU350335   ................................-.......T.............................
GB-4_O.aries_Z35240             ................................-.....................................
GB-5_O.aries_Z35233             ................................-.....................................
GB-6_O.aries_Z35267             ................................-.....................................
GB-7_O.aries_musimon_GU350328   ................................-.......T.............................
IRQ52513                        ................................-.......T.............................
IRQ52013                        ................................-.......T.............................
GB-8_O.aries_musimon_AF039579   ................................-.......T.............................
GB-9_O.aries_musimon_AY091487   ................................-.......T.............................
GB-10_O.aries_musimon_HM236184  ................................-.......T.............................
IRQ15013                        ................................-.......T.............................
IRH000213                       ................................-.......T.............................
IRQ071212_A                     ................................-.......T.............................
IRQ059412                       ................................-.......T.............................
GB-11_O.aries_ophion_KF312238   ................................-.......T.............................
GB-12_O.aries_Z35249            ................................-.....................................
GB-13_O.aries_AB006801          .............G.......A.......TC.C.......T...C......A..................
IRQ02713_C.capreolus            .............................TC.C.......T..........A..................
IRQ039412_C.elaphus             .............................TC.C.......T..........A......A.......A.T.
Clustal Consensus               ************* ******* *******  * ******* *** ****** ****** ******* * *

GB-1_O.aries_Z35264             TTTTGGGG-ATGCTTGGACTCAGCTATGGCCGTCTG-AGGCCCCGACCCGGAGCATGAATTGTAGCTGGA
GB-2_O.aries_Z35258             ........-...........................-.................................
GB-3_O.aries_musimon_GU350335   ........-...........................-......T..........................
GB-4_O.aries_Z35240             ........-...........................-.................................
GB-5_O.aries_Z35233             ........-...........................-...................AG............
GB-6_O.aries_Z35267             ........-...........................-.................................
GB-7_O.aries_musimon_GU350328   ........-...........................-.................................
IRQ52513                        ........-...........................-.................................
IRQ52013                        ........-...........................--A.G.............................
GB-8_O.aries_musimon_AF039579   ........-...........................--A.G.............................
GB-9_O.aries_musimon_AY091487   ........-...........................-.................................
GB-10_O.aries_musimon_HM236184  ........-...........................-.................................
IRQ15013                        ........-...........................-.................................
IRH000213                       ........-...........................-.................................
IRQ071212_A                     ........-...........................-.................................
IRQ059412                       ........-...........................-...................A.............
GB-11_O.aries_ophion_KF312238   ........-...........................-...................A.............
GB-12_O.aries_Z35249            ........-...........................-.................................
GB-13_O.aries_AB006801          G.C.....-...................A..A....-......T.............T...........G
IRQ02713_C.capreolus            ........G.........................AA-...................A.............
IRQ039412_C.elaphus             ...G....G...............A...A.......G...T....T........................
Clustal Consensus                *  **** *************** *** ** **     * ** * **********  ***********

GB-1_O.aries_Z35264             CTTAACTGCATCTTGAGCATCCTCATAAT
GB-2_O.aries_Z35258             .............................
GB-3_O.aries_musimon_GU350335   .............................
GB-4_O.aries_Z35240             .............................
GB-5_O.aries_Z35233             .............................
GB-6_O.aries_Z35267             .............................
GB-7_O.aries_musimon_GU350328   ......................C......
IRQ52513                        ......................C....--
IRQ52013                        ......................C...---
GB-8_O.aries_musimon_AF039579   ......................C......
GB-9_O.aries_musimon_AY091487   ......................C......
GB-10_O.aries_musimon_HM236184  ......................C......
IRQ15013                        ......................C...---
IRH000213                       ......................C...---
IRQ071212_A                     .........C............C...---
IRQ059412                       ......................C....--
GB-11_O.aries_ophion_KF312238   .............................
GB-12_O.aries_Z35249            .............................
GB-13_O.aries_AB006801          .....................AC......
IRQ02713_C.capreolus            ......................C...---
IRQ039412_C.elaphus             ......................C.....-
Clustal Consensus               ********* ***********  ***
```
