# Supplementary material for: Identification of Ixodes ricinus blood meals using an automated protocol with high resolution melting analysis (HRMA) reveals the importance of domestic dogs as larval tick hosts in Italian alpine forests
Source: Parasit Vectors. 2016 Dec 12;9:638. doi: 10.1186/s13071-016-1901-y (PMC5154095; doi:10.1186/s13071-016-1901-y)
Supplement: Additional file 3: Figure S1. — Sequence alignment of mtDNA control region amplicons generated with the Muroidea primer set. (PDF 307 kb) [file 13071_2016_1901_MOESM3_ESM.pdf]

Additional file 2.

Sequence alignment of mtDNA *control region* amplicons generated with the primer set HRM_Rod [11]. IRH control samples sequence; IRQ questing nymphs sequence. Sequences 1-13: *Apodemus* spp.; 9 -13 *Apodemus* spp. amplicons (as resulting from BLASTn search) with deletions and mutations resulting in a deviating melting pattern leading to misidentification by HRMA as *M. glareolus*; 14-15: *M. glareolus* amplicons. Reported are also melting temperature, in bold refer to QIAgen extracted samples, while normal to ThermoScientific extracted samples.

```
                              10        20        30        40        50        60        70
                     ....|....|....|....|....|....|....|....|....|....|....|....|....|....|
1-IRH_A.flavicollis  CTGGTTCTTACTTCAGGGCCATCAAATGCGTTATC-GCCCATACGTTCCCCTTAAATAAGACATCTCG-A
2-IRQ                                   ................-................................-.
3-IRQ                                   ................-................................-.
4-IRQ                                   ................-................................-.
5-IRQ                                   ................-................................-.
6-IRQ                                   ................-................................-.
7-IRQ                                   ................-................................-.
8-IRQ                                   ................-................................-.
9-IRQ                                   ................C................................-.
10-IRQ                                  ................-................................-.
11-IRQ                                  ...TG...........-................................-.
12-IRQ                                  ...TG...........-...............................A-.
13-IRQ                                  ...TG...........-...............................AG.
14-IRH_M.glareolus                      ...TC....TT.....-.T..............................-.
15-IRQ                                  ...TC....TT.....-.T..............................-.
Clustal Consensus                       ***  ****  ***** * *****************************  *

                             80        90       100       110       120       130       140
                     ....|....|....|....|....|....|....|....|....|....|....|....|....|....|
1-IRH_A.flavicollis  TGGTACGGGTCTAATCAGCCAAGACTTC-CATAACTGTGGTCTCGGGCAGTTGGTATTTTTTTATTTTTA
2-IRQ                ............................-.........................................
3-IRQ                ............................-.........................................
4-IRQ                ............................-.........................................
5-IRQ                ............................-.........................................
6-IRQ                ............................-.........................................
7-IRQ                ............................-.........................................
8-IRQ                ............................-.........................................
9-IRQ                ..................A.........-.........................................
10-IRQ               ............................-........---..............................
11-IRQ               .....T......................-........---......................-.......
12-IRQ               .................A..........-........---...T..........................
13-IRQ               .........................C..-........---......A.......................
14-IRH_M.glareolus   ....................C.TG.C.AA.................................A.....CG
15-IRQ               ....................C.TG.C.AA.................................A.....CG
Clustal Consensus    ***** ***********  * *  * *  ********   *** ** *************** *****

                            150       160       170
                     ....|....|....|....|....|....|....|.
1-IRH_A.flavicollis  GGATGCTGTGACTCAGCATAGCCGTCAAGGCATGAA
2-IRQ                ................
3-IRQ                ................
4-IRQ                ................
5-IRQ                ................
6-IRQ                ................
7-IRQ                ................
8-IRQ                ................
9-IRQ                ................
10-IRQ               ................
11-IRQ               ................
12-IRQ               ................
13-IRQ               .....T..........
14-IRH_M.glareolus   .....G.A.C......
15-IRQ               .....G.A.C......
Clustal Consensus    ***** * * ******
```

Forward primer

Reverse primer

Sample	T_m (°C)
1-IRH_A.flavicollis	**81.9**
2-IRQ	**81.90**
3-IRQ	**81.50**
4-IRQ	82.06[a]
5-IRQ	82.20
6-IRQ	82.44
7-IRQ	82.40
8-IRQ	82.46
9-IRQ	82.00
10-IRQ	**81.30**
11-IRQ	81.10
12-IRQ	81.34
13-IRQ	82.00
14-IRH_M.glareolus	**81.12**
15-IRQ	**81.00**

[a] despite no mutation this sample showed a lower melting temperature than reference samples, possibly because of abnormal condition in the HRMA run.
